# What can livestock breeders learn from conservation genetics and *vice versa*?

**DOI:** 10.3389/fgene.2015.00038

**Published:** 2015-02-10

**Authors:** Torsten N. Kristensen, Ary A. Hoffmann, Cino Pertoldi, Astrid V. Stronen

**Affiliations:** ^1^Section of Biology and Environmental Science, Department of Chemistry and Bioscience, Aalborg UniversityAalborg, Denmark; ^2^Department of Zoology and Department of Genetics, Bio21 Institute, The University of MelbourneMelbourne, VIC, Australia; ^3^Aalborg ZooAalborg, Denmark

**Keywords:** effective population size, inbreeding, indigenous breeds, genetic rescue, genomics, selection

## Abstract

The management of livestock breeds and threatened natural population share common challenges, including small effective population sizes, high risk of inbreeding, and the potential benefits and costs associated with mixing disparate gene pools. Here, we consider what has been learnt about these issues, the ways in which the knowledge gained from one area might be applied to the other, and the potential of genomics to provide new insights. Although there are key differences stemming from the importance of artificial versus natural selection and the decreased level of environmental heterogeneity experienced by many livestock populations, we suspect that information from genetic rescue in natural populations could be usefully applied to livestock. This includes an increased emphasis on maintaining substantial population sizes at the expense of genetic uniqueness in ensuring future adaptability, and on emphasizing the way that environmental changes can influence the relative fitness of deleterious alleles and genotypes in small populations. We also suspect that information gained from cross-breeding and the maintenance of unique breeds will be increasingly important for the preservation of genetic variation in small natural populations. In particular, selected genes identified in domestic populations provide genetic markers for exploring adaptive evolution in threatened natural populations. Genomic technologies in the two disciplines will be important in the future in realizing genetic gains in livestock and maximizing adaptive capacity in wildlife, and particularly in understanding how parts of the genome may respond differently when exposed to population processes and selection.

## INTRODUCTION

The effective population size, Ne, is a measure of fundamental importance for understanding the potential of species and populations to evolve and adapt to natural and artificial selection pressures. Quantitative genetic theory predict that Ne is positively associated with the level of additive genetic variation and that the capacity of a population to respond to selection depends on the level of genetic variation for the trait(s) undergoing selection (see Falconer and Mackay, 1996). However, the association between Ne, genetic variation and evolutionary potential is complex and depends on factors such as the number of loci underlying a trait, the presence of dominance or epistasis, the effects of new mutations, and selection mode and intensity (reviewed in Willi et al., 2006).

In natural populations, there are numerous examples showing that a small Ne can reduce adaptive potential as a consequence of reduced genetic variation (e.g., Markert et al., 2010; Siol et al., 2010; Strasburg et al., 2011; Gossmann et al., 2012; Phifer-Rixey et al., 2012) and also as a consequence of a reduction in fitness due to inbreeding depression (Mattila et al., 2012; Hoffman et al., 2014). However, some small populations remain capable of adapting through evolutionary changes to shifting environmental conditions (references in Merilä, 2014).

Populations of domestic animals with a small Ne can also exhibit reduced genetic variation compared to ancestral ones (Ghafouri-Kesbi et al., 2008; Kim et al., 2013; Freedman et al., 2014; Quaresma et al., 2014). For example Freedman et al. (2014) found that the domestication process of dogs resulted in reduced genomic variation consistent with at least a 16-fold reduction in population size. On a shorter time scale Kim et al. (2013) showed that selection in US Holstein dairy cattle during the last 50 years has led to increased autozygosity across the genome. Inbreeding depression due to low Ne occur in domestic animal populations as well as in natural populations, decreasing milk yield and fat and protein content in the milk in dairy cattle and growth rates in sheep (Croquet et al., 2006; Pedrosa et al., 2010) and increasing the incidence of diseases such as mastitis in dairy cattle (Croquet et al., 2006; Sørensen et al., 2006).

However, a small Ne in domesticated animal populations helps produce phenotypic uniformity within populations (and even more so in domesticated crops) that results in products that can be more easily processed and marketed (Allard, 1999; Aslam et al., 2012; Janhunen et al., 2013). Intense directional artificial selection (and strong selection responses) in livestock is partly attained by use of a few selected animals, with superior genetic profiles, making the genetic contribution of animals in a population highly skewed. This is one reason why Ne below 100 is observed within many intensively managed modern breeds (Leroy et al., 2013). Despite low Ne, large and ongoing genetic gains for production traits are typically achieved in commercial livestock (Hill and Kirkpatrick, 2010).

Although the implications of small Ne in natural populations and domestic breeds are somewhat different, there are insights to be gained from combining knowledge of these disparate areas. Genetic studies on small and fragmented populations in nature, such as island populations or populations at the brink of extinction, provide ideas and concepts that could be applied to the management of small domestic populations. This includes applying genetic rescue to boost the adaptive potential of small populations, and using genotype by environment interactions to ensure populations maintain a high fitness across environments (Ingvarsson, 2001; Vilá et al., 2003; Tallmon et al., 2004; Armbruster and Reed, 2005; Edmands, 2007; Adams et al., 2011; Reed et al., 2012). On the other hand, animal and plant breeders have shown how deep pedigrees, detailed phenotypic information, large sample sizes and reproductive technologies can be effectively used to meet genetic challenges in small populations. Information from pedigrees is already being applied to natural populations of birds and mammals where individuals can be tracked and followed across generations (e.g., Reid et al., 2006; Nielsen et al., 2012; Hedrick et al., 2014), and there is also potential to apply other approaches from livestock management to small and threatened natural populations.

While Ne is important from a genetic perspective, the census size of domestic and natural populations is obviously also important for predicting extinction risk. Populations at a small census size are more likely to go extinct due to factors such as demographic and environmental stochasticity even if genetic considerations are not taken into account. However, in this paper we focus only on the consequences of low Ne, providing examples of genetic rescue in natural populations, and considering prospects of using genomics as a tool that could advance long-term conservation management in both natural and domestic populations.

## CONSEQUENCES OF LOW Ne – INBREEDING AND EVOLUTIONARY POTENTIAL

**FIGURE 1:**
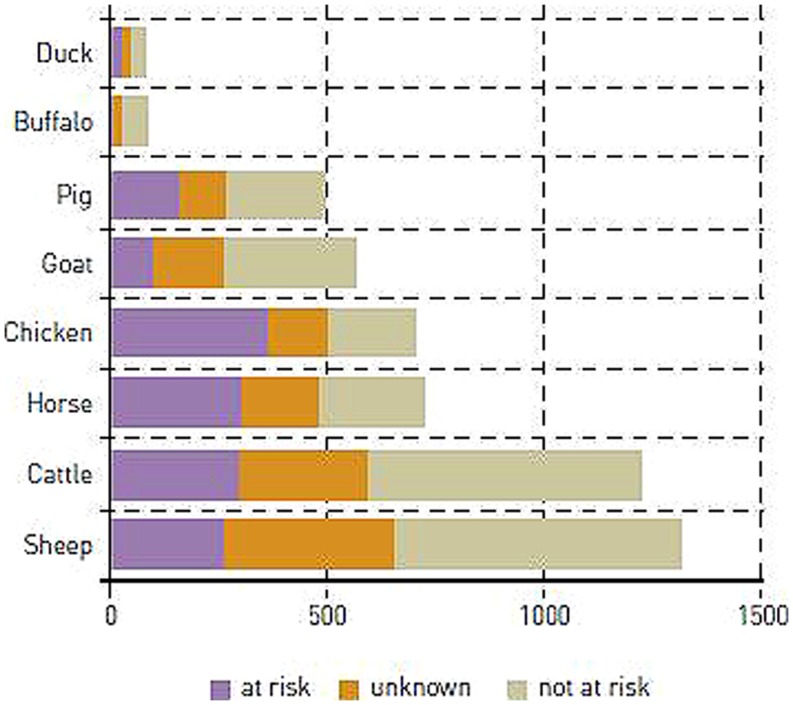
**Estimated numbers of domestic animal breeds at risk worldwide (FAO Livestock report, 2005)**.

Most domestic breeds are small with Ne typically counted in tens or a few 100s and some of them are threatened by extinction (Hill and Kirkpatrick, 2010; Leroy et al., 2013; **Figure 1**). Likewise a large and increasing number of wild populations are threatened and also have low Ne (references in Frankham et al., 1998; IUCN, 2014). Populations with small Ne are prone to inbreeding and loss of genetic variation due to genetic drift (Falconer and Mackay, 1996; Willi et al., 2006).

### INBREEDING

**FIGURE 2:**
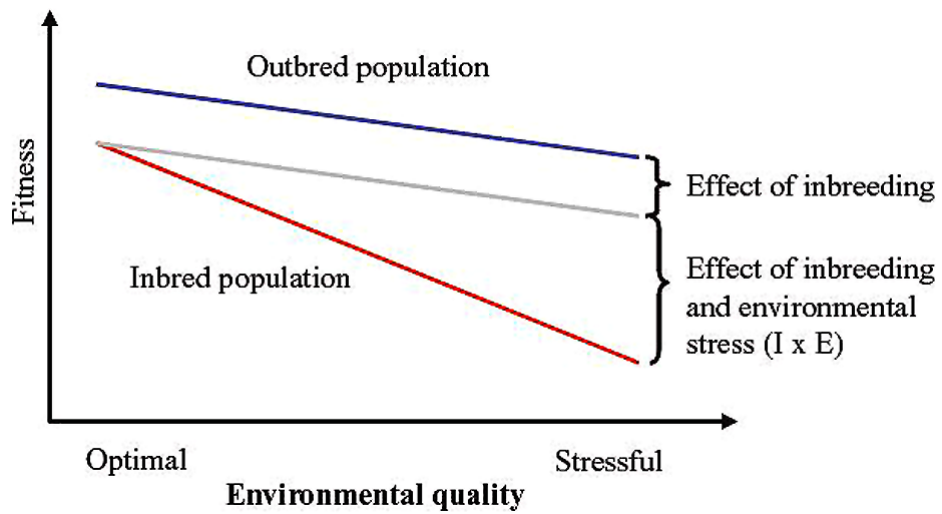
**Schematic illustration of fitness effects of inbreeding by environment interactions.** Assuming the effect of inbreeding is independent of the environment, the reduction in fitness as a result of reduced environmental quality will be equal for outbred and inbred populations. The blue and gray lines illustrate fitness of an outbred and an inbred population, respectively, in the absence of inbreeding by environment interactions. Inbreeding depression is, however, often more severe under stressful environmental conditions. Thus, the red line illustrates fitness of an inbred population taking into account the effect of inbreeding by environment interactions (redrawn from Kristensen et al., 2010).

Mating of related individuals is unavoidable in populations of finite sizes but occurs also when there is preferential mating of relatives in large populations. Inbreeding leads to a reduced number of heterozygotes and ultimately to complete homozygosity in the genome (Falconer and Mackay, 1996). Inbred individuals typically have lower fitness (inbreeding depression) although effects are highly trait and population specific (Frankham et al., 1998; Kristensen and Sørensen, 2005). Frankham et al. (1998) summarized estimates of inbreeding depression across different components of fitness in 15 domesticated and non-domesticated animals and plant species, and concluded that a 25% increase in inbreeding reduced mean fitness of inbred compared to outbred individuals by 15%. Levels of inbreeding depression are on average higher for fitness-related traits compared to morphological or behavioral traits (DeRose and Roff, 1999; Willi et al., 2006). Further there is evidence that inbred individuals are less robust when exposed to stressful environmental conditions compared to outbred individuals (i.e., inbreeding effects are exacerbated by stressful conditions, resulting in inbreeding by environment interactions; Armbruster and Reed, 2005; Reed et al., 2012; **Figure 2**). When estimates of inbreeding depression from Frankham et al. (1998) are separated into estimates from domestic and non-domesticated species, it appears that inbreeding depression may be somewhat less pronounced in the former group (12 versus 17%). Although this needs further investigation, this difference might reflect the relatively benign and less variable environments experienced by domestic animals. The environmental dependency of inbreeding depression has been shown in numerous studies in natural populations (e.g., Jimenez et al., 1994; Hauser and Loeschcke, 1996; Keller et al., 2002; Szulkin and Sheldon, 2007) but surprisingly mostly in older literature on animal and plant breeding (e.g., Finlay, 1963; Hull, 1963).

There is evidence in wild as well as domestic populations that recessive deleterious alleles can be purged with inbreeding (Hedrick and Kalinowski, 2000; Crnokrak and Barrett, 2002; Charlesworth, 2009). This results in inbreeding depression being diminished in *Drosophila* and bird populations with a long history of inbreeding (Swindell and Bouzat, 2006; Laws and Jamieson, 2011). Purging, however, can be environment-specific; thus *Drosophila* studies have shown that purging performed in one environment might not be efficient in reducing inbreeding depression in another environment (Bijlsma et al., 1999; Dahlgaard and Hoffmann, 2000; Mikkelsen et al., 2010).

Both environment-specific purging and inbreeding by environment interactions suggest that an inbred population that does not currently suffer from inbreeding depression is nevertheless likely to do so if the environment changes and particularly if it becomes more stressful. This is expected to contribute to the extinction risk in small natural populations facing environmental shifts including dramatic climate changes. Liao and Reed (2009) determined that inclusion of fitness effects stemming from inbreeding by environment interactions reduced persistence times of natural populations by 17.5–28.5% across a wide range of scenarios. This might be an overlooked reason for poor performance of domestic livestock moved across areas where production systems differ, such as dairy cattle breeds exported from temperate to tropical climates and *vice versa* (Zwald et al., 2003).

Genomic tools will increase our understanding of the genetic architecture of inbreeding depression in natural and domestic populations (Charlesworth and Willis, 2009; Kristensen et al., 2010; Ouborg et al., 2010). Genomics is already used to accurately estimate levels of inbreeding, detect loci that contribute significantly to inbreeding depression, and dissect the history of inbreeding in a population (old or recent; Charlesworth, 2009; Purfield et al., 2012; Curik et al., 2014; Pertoldi et al., 2014). Genomic tools should also help untangle the population and trait specific nature of inbreeding effects by identifying the nature of interactions between identified loci and their genetic background. So far genomic tools used to dissect the genetics of inbreeding have mainly been applied to model organisms and livestock but genomic resources are also rapidly emerging for wild animals and plants (Ouborg et al., 2010; Ekblom and Galindo, 2011). Dissecting the genomic architecture of inbreeding in natural populations is helped if there are genomic resources available for a related species; this facilitates assembly and functional annotation (Ekblom and Galindo, 2011). Hence a mapped and annotated cattle or chicken genome can facilitate studies on small and inbred natural populations of closely related mammal and bird species (Romanov et al., 2009).

### GENETIC DRIFT AND LOSS OF GENETIC VARIATION

Genetic drift represents the random change of allele frequencies over generations due to finite population size, and a small Ne is expected to increase the rate of genetic drift and associated loss of genetic variation across generations as alleles become fixed (Wright, 1929; Falconer and Mackay, 1996; Willi et al., 2006; Merilä, 2014). These theoretical predictions are generally supported by empirical data from model organisms and wildlife (and to a lesser degree in livestock) showing less genetic variation and selection responses in populations with small Ne (Jones et al., 1968; Weber, 1990; Caballero et al., 1991; Kristensen et al., 2005; Hill and Kirkpatrick, 2010; Olson-Manning et al., 2012; Kim et al., 2013). The high heterozygosity values sometimes reported in domestic breeds despite low Ne may, at least in part, result from biased selection of hypervariable microsatellite markers in chromosome regions not under selection (Taberlet et al., 2008). Kim et al. (2013) provide support for this hypothesis showing that Holstein dairy cattle selected intensively for increased milk yield do have increased overall autozygosity when assessed across the genome; this is likely a consequence of both genetic drift and selection.

What practical implications do expected reductions in genetic variation in small populations have for the management of small breeds? Breeding in commercial dairy cattle breeds like Holstein Frisian and Jersey with Ne’s below 100 has been very efficient and no apparent signs of selection plateaus have been seen (Chikhi et al., 2004; Sørensen et al., 2005; Hill and Kirkpatrick, 2010; Leroy et al., 2013). With very high selection intensity, most traits can be changed through directional selection (Hill and Kirkpatrick, 2010) and genetic variation may remain relatively stable within the time frames typically considered by a breeding company or an individual farmer. Many domestic species have long generation length, and intense selection within domestic breeds has (from an evolutionary point of view) not been practiced for long. The breed concept is not more than approximately 200 years old, and reproductive technologies enabling intense selection has only been in common use since the 1960s (Taberlet et al., 2011).

Genomic selection applied to animal breeding will reduce generation intervals which is one reason for expected increased genetic gain using this technique (Schaeffer, 2006; Meuwissen, 2007). However, assuming that the high level of genetic variation observed in some domestic breeds despite low Ne is partly a consequence of the long generation length (genetic variation is simply not lost yet), genomic selection may speed up loss of genetic variation. It is estimated that generation intervals in dairy cattle will be reduced by 50% in the future because individuals will have breeding values at birth (in contrast to the situation where an individual’s performance has to be assessed first; Blasco and Toro, 2014). Thus genomic selection could potentially double the speed at which genetic variation is lost within breeds, even though genomic selection can also be used to optimize heterozygosity (see, e.g., Pedersen et al., 2009; Pertoldi et al., 2014).

**BOX 1. General recommendations for conservation of populations of species with conservation concerns.**

*Scenario 1:* A genetically unique population that is highly adapted to local conditions and has high levels of genetic variation. *Recommendation:* If Ne >500–1000 and not decreasing there may be no need to change management strategies. *Conservation priority:* High.

*Scenario 2:* A genetically unique population (that is) highly adapted to local conditions but with limited genetic variation. *Recommendation:* Despite currently being successful in its environment some gene flow from populations is recommended to increase Ne and genetic variation. *Conservation priority:* High.

*Scenario 3:* A population that is unique but maladapted and has high levels of genetic variation. *Recommendation:* Characterize why this population is maladapted and start to select (if the population is managed) for increased local adaptation. Gene flow from populations adapted to similar environments is recommended if the population does not respond to selection. *Conservation priority:* Intermediate.

*Scenario 4:* A population that is locally adapted, but not genetically unique and with low levels of genetic variation. *Recommendation:* Management should prioritize increasing Ne and genetic variation. Gene flow from populations adapted to similar environments is recommended. *Conservation priority:* Low assuming that there are other conspecific populations available.

*Scenario 5:* A population that is not unique, maladapted, and with low levels of genetic variation. *Recommendation:* Gene flow from populations adapted to similar environments is needed. *Conservation priority:* Low.

### Ne RECOMMENDATIONS

Rules of thumb that have influenced management of domestic and wild populations since the early 1980s state that (1) an Ne of at least 50 is needed for avoiding inbreeding depression in the short term (five generations), and (2) an Ne of at least 500 are sufficient to retain long-term evolutionary potential (Franklin, 1980; Soulé, 1980; Franklin and Frankham, 1998). It has been suggested that these numbers are much too low (Willi et al., 2006; Frankham et al., 2014) for several reasons, including the fact that they are not based on realistic N/Ne ratios, they do not take into account inbreeding depression and that environments are changing at an unprecedented speed. The value of Ne recommendations in a conservation management context has been the subject of extensive discussion (see, e.g., Willi et al., 2006; Jamieson and Allendorf, 2012; Frankham et al., 2014). Such recommendations can be criticized because the potential to adapt (1) depends on the type of environmental change and the type of traits, and (2) the evolutionary potential of small populations is compromised by processes that do not directly alter genetic variation such as suboptimal environmental conditions (which may lower heritability estimates due to increased environmental variance), inbreeding depression (Willi et al., 2006), and inbreeding by environment interactions (Armbruster and Reed, 2005; Reed et al., 2012). Despite these limitations, recommendations remain useful because the alternative might be unscientific conservation decisions made at the political and bureaucratic levels (Frankham et al., 2014; **Box 1**).

For domesticated animals, Leroy et al. (2013) found that the large majority of domestic breeds have Ne’s below 500 when estimated as the increase in homozygosity over generations by measuring identity by descent probabilities. Thus Ne estimates were below 500 in all but a few of the 20 cattle breeds, 40 sheep breeds, 20 horse breeds, and 60 dog breeds investigated, and several had Ne estimates below 50 (Leroy et al., 2013). The low Ne estimates observed in livestock are mirrored in many wildlife species and populations, although precise estimates are difficult to obtain and tend to vary depending on the method employed (Frankham, 1995; Luikart et al., 2010; Leroy et al., 2013). According to the International Union for Conservation of Nature (IUCN, 2014), more than 22,000 species are currently threatened in nature. Effective population sizes of these species are rarely known, but assuming a Ne/N ratio of 0.1 the huge majority of these species have effective population sizes below 500 individuals. Thus these numbers match findings in livestock breeds.

However, these estimates may be revised as more accurate estimates of Ne based on genome-wide markers emerge. A recent study on Chinook salmon (*Oncorhynchus tshawytscha*) estimated Ne from RAD-seq data and found surprisingly high Ne estimates given the census size of these populations (Larson et al., 2014). This approach will also be valuable in Ne estimates for livestock and possibly shed light on the paradox that many commercial breeds have low Ne (typically based on pedigree data) but high and continued selection responses (Hill and Kirkpatrick, 2010).

With Ne being low for many breeds and natural populations, a challenge is to take steps to ensure that Ne is maintained or even increased. Equalizing sex ratios of those animals contributing to the next generation, and reducing variation in the number of offspring, directional selection, inbreeding and variation in Ne across generations are all important for increasing Ne (or diminishing its further reduction; Charlesworth, 2009). In many European countries farmers are subsidized for keeping indigenous breeds. Ways to encourage stabilization or increases in breed Ne would be to support initiatives such as allowing more males to contribute to the next generation to equalize sex ratios, to include cryopreserved genetic material in the breeding plan (Sonesson et al., 2002), or by controlling inbreeding in the herd efficiently such as by the use of software programs (Sørensen et al., 2008). However, increasing Ne is a daunting task in a small population where there is no possibility of increasing genetic variation through immigration. The harmonic means of Ne across generations describe the impact of fluctuations in population size on overall Ne (Caballero, 1994; Falconer and Mackay, 1996). A population that has been through a genetic bottleneck will suffer long lasting consequences in terms of reduced Ne despite the fact that the population size might have increased following the bottleneck (Charlesworth, 2009). Genetic rescue of a population or breed might then be required as discussed below.

According to Ne recommendations mentioned above, the long-term evolutionary potential for the majority of livestock breeds and threatened wild species is expected to be severely diminished. The number of generations required to reach selection plateaus in small populations obviously depends on numerous factors such as the level of standing genetic variation for the trait in question, number of loci contributing to the variation, and the selection intensity. Results from selection experiments are diverse but suggest that selection plateaus are typically reached in less than 30 generations in mice and *Drosophila* (references in Falconer and Mackay, 1996). With a typical 3–4 years generation interval (defined as the average age of parents when offspring are born) for cattle, this corresponds to ∼90–120 years of selection. Still there is no evidence suggesting selection plateaus in the majority of commercial livestock breeds (Hill and Kirkpatrick, 2010). Genomics data on livestock are likely to help understand how selection has shaped genetic variation in populations, such as by enabling more accurate Ne estimates and pinpointing which parts of the genome have been under selection and remain variable.

## CONSEQUENCES OF LOW Ne IN SMALL INDIGENOUS LIVESTOCK BREEDS

Many indigenous livestock breeds have low Ne and several are considered threatened, endangered or already extinct (Hoffmann, 2013; Leroy et al., 2013; DAD-IS, 2014; **Figure 1**). Intense and structured directional selection is typically not performed in these breeds, and many of them have been through more extreme bottlenecks compared to the commercial breeds, with documented low levels of genetic variation (Melis et al., 2012; Herrero-Medrano et al., 2014; Pertoldi et al., 2014). Accordingly, they are more likely to suffer from evolutionary constraints, inbreeding and drift load due to low Ne compared to modern commercial breeds. From an evolutionary point of view this has two obvious and immediate consequences of relevance for the management of indigenous breeds with small Ne: (1) in breeds with no remaining genetic variation, evolution is constrained no matter how intense the selection pressure, unless it involves removal of newly arisen deleterious alleles, and (2) even if genetic variation is present, only small responses to selection are expected. This is because when Ne decreases, the impact of genetic drift increases and loci under selection start to behave as neutral when selection coefficients become equal or smaller than 1/[2Ne] (Wright, 1931). Hence, to prevent the loss of rare beneficial alleles by genetic drift in small populations, stronger selection is required.

There will sometimes be good cultural, historical and also genetic reasons for managing indigenous (and commercial) breeds as separate and closed breeds. However, this will rarely be the most efficient management practice if the goal is to conserve breeds, adaptive genetic variation and the scope for local adaptation in the long run. *In situ* conservation is sometimes referred to as the golden standard in the management of domestic breeds. We argue that for *in situ* conservation to be efficient in maintaining and generating locally adapted breeds, Ne should be increased so that it counts 100s and not tens of animals. Until this goal is achieved, what matters from a genetic perspective is to increase genetic variation so that evolution is not constrained. The generation of new mutations is a very slow process, and genetic rescue by bringing in new variation from other populations will often be the only option (see **Box 1** for recommendations regarding the management of small populations).

## LOW Ne AND MALADAPTATION

In the literature it is sometimes taken for granted that a population in the wild or an indigenous livestock breed is locally adapted to the habitat or geographical region in which it is present. Although some well-documented cases exists, especially from the tropics (Bayer et al., 1987; Ayantunde et al., 2002; references in Hoffmann, 2010, 2013), it is often not known whether a given phenotype represent an adaptation to the local environment (Hoffmann, 2013). This issue also applies to natural populations where there is often good evidence for local adaptation (Dobzhansky, 1956) but also many cases where it does not seem to occur (references in Crespi, 2000). Thus proper, standardized and continuous phenotypic and genotypic characterization according to guidelines such as those suggested by FAO (2011, 2012) is highly recommended.

Maladaptation can have many genetic causes, including mutation, inbreeding, random genetic drift, gene flow leading to breakdown of co-adapted gene complexes, heterozygote advantage and pleiotropy (Crespi, 2000). Space does not allow reviewing these causes in detail but we discuss some aspects relevant for the management of domestic breeds and small natural populations (see also **Box 1**).

Gene flow and genetic drift might prevent or disrupt local adaptation and lead to maladaptation or outbreeding depression. Due to genetic drift, genes of adaptive value may behave neutrally, potentially leading to maladaptation in small populations. Gene flow may prevent local adaptation (Stearns and Sage, 1980), but this depends on whether gene flow is constrained by geographical distance or the environment, and cases of maladaptive gene flow in wild populations are relatively uncommon (Sexton et al., 2014). Introgression may have deleterious effects if there is outbreeding depression, however, the probability of outbreeding depression in crosses between two populations of the same species appears to be low for populations with the same karyotype, isolated for <500 years, and that occupy similar environments (Frankham et al., 2011). These are important aspects when considering cross-breeding as an option in domestic breeds. Amador et al. (2014) present two genomic selection strategies, using genome-wide DNA markers, to recover the genomic content of the original endangered population from admixtures. Such tools will be useful in the conservation of domestic populations where crossing between breeds has occurred intentionally or unintentionally and it is worthwhile recovering the breeds.

## GENETIC RESCUE – EXAMPLES FROM THE WILD

The merits and challenges of genetic rescue – augmenting genetic variation and limiting inbreeding depression – have been evaluated for over a decade in wildlife biology (e.g., Ingvarsson, 2001; Vilá et al., 2003; Tallmon et al., 2004; Edmands, 2007; Adams et al., 2011). The lessons learned may offer important insights for conservation of threatened livestock, where many breeds are facing threats parallel to those of wild species including inbreeding and rapid loss of genetic variation (Kristensen and Sørensen, 2005; Leroy et al., 2013).

Examples of genetic rescue include the arrival of new wolves (*Canis lupus*) into the isolated and highly inbred populations on Isle Royale, MI, USA (Adams et al., 2011) and the Scandinavian Peninsula (Vilá et al., 2003; Hagenblad et al., 2009). These have led to a subsequent increase in genetic diversity, and potentially in fitness although the latter is challenging to measure in wild populations (Ingvarsson, 2001, 2002), possibly short-lived (Hedrick and Fredrickson, 2010; Hedrick et al., 2014), and may be masked by environmental factors such as availability of food and space (Adams et al., 2011).

A consideration for relocation programs in augmenting genetic diversity has been the introduction of donor animals from environments as similar as possible to the new location (e.g., Hedrick and Fredrickson, 2010), to help avoid maladaptation/outbreeding depression. This can occur when donor and recipient populations are adapted to different environmental conditions and the resulting hybrid offspring are ill-suited to either habitat (Templeton, 1994). Examples reported in the past include translocations of Arabian oryx (*Oryx leucoryx*; Marshall and Spalton, 2000), as well as ibex (*Capra ibex*) from Sinai and collared lizards (*Crotaphytus collaris*) from the US Ozark mountains (Templeton, 1994). However, the relationship between population divergence and hybrid fitness is highly variable among taxa and may be difficult to predict without experimental crosses, which, although recommended, are not always feasible in particular for long-lived species with long generation times (Edmands, 2007 and references therein; Hedrick and Fredrickson, 2010).

In some instances, genetic rescue occurs naturally and without human intervention or knowledge, and is later confirmed by genetic investigations (e.g., Scandinavian wolves; Ingvarsson, 2002; Vilá et al., 2003; Hagenblad et al., 2009). In these situations, the arrival of the rescuing individual(s) typically poses no ethical or conservation dilemma. In contrast, there is discussion concerning the merits and ethical implications of human-mediated genetic rescue. The small and isolated wolf population on Isle Royale in Lake Superior, USA, represents a valuable illustration (Vucetich et al., 2012, 2013; Cochrane, 2013; Mech, 2013). Wolves arrived unassisted on the island by crossing the ice, and the population has experienced bottlenecks, in part owing to suspected human introduction of parvovirus in dogs that caused a crash in the wolf population (Peterson et al., 1998). Inbreeding is reported to have caused problems with bone malformations, which could limit movement and potentially the ability to hunt large prey such as moose (*Alces alces*) and reduce general life expectancy (Räikkönen et al., 2009). The arrival of a more recent immigrant appears to have produced a selective sweep and genetic rescue of the population, but isolation and environmental conditions (such as reduced ice cover with climate change and thus reduced chance of new immigrants) pose continuing long-term challenges (Hedrick and Fredrickson, 2010; Adams et al., 2011).

At times, it may be necessary to crossbreed with individuals from another relatively similar population to augment genetic diversity. In such situations there may be no ideal solution for the introduction of new genetic material. Genetic rescue of the Florida subspecies of panther (*Puma concolor coryi*) implied relocating individuals from the closest wild population, a subspecies from Texas (*Puma concolor stanleyana*), which was a controversial decision (Pimm et al., 2006; Hedrick and Fredrickson, 2010; Johnson et al., 2010). The remnant Florida population suffered from several problems believed to be associated with genetic drift and inbreeding, such as undescended testicles and morphological abnormalities (reviewed in Pimm et al., 2006; Hedrick and Fredrickson, 2010). The relocation appears to have improved the survival, genetic diversity and range of the Florida panther and augmented the population, at least for the moment (Pimm et al., 2006; Hedrick and Fredrickson, 2010; Johnson et al., 2010).

Management decisions will often have to be made with incomplete knowledge of all relevant scientific data, and requires explicit acknowledgment of the ethical norms that are guiding principles in conservation biology (Soulé, 1985). The social and biological sciences are therefore both important, as is the acknowledgment that human intervention may play an essential and often necessary role. Additionally, Templeton (1994) and Weeks et al. (2011) recommend prioritizing genetic diversity and the potential for evolutionary change, and note that conservation efforts should aim to preserve processes such as evolution rather than specific genetic variants. In this context, human-induced fragmentation and subsequent genetic drift may have had a major influence on wildlife such as Texas and Florida panthers that in the past likely exchanged genes via intermediary populations (Hedrick and Fredrickson, 2010).

## GENETIC RESCUE IN DOMESTIC BREEDS

A situation parallel to these wild populations, involving a small population with few breeders, and ensuing risks of reduced fitness and long-term survival, is the endangered Norwegian Lundehund, a Spitz breed native to coastal Norway where it was historically used to hunt puffins (*Fratercula arctica*). The remaining individuals have extremely low genetic diversity and are highly inbred (Melis et al., 2012; Pfahler and Distl, 2013). Efforts are currently underway to evaluate similar Nordic Spitz breeds for possible cross-breeding^1^. Although the genetic background for the condition(s) remains unknown, the Lundehund is affected by serious gastrointestinal problems that seem particularly prevalent for this breed (Landsverk and Gamlem, 1984; Berghoff et al., 2007; Qvigstad et al., 2008) and for which cross-breeding may be beneficial. Whereas cross-breeding may alter the breed’s morphology and behavior, there appears to be no alternative means of increasing genetic diversity.

Native livestock species may be well-adapted to their regions and be of historical and economic importance (Joost et al., 2007; Pariset et al., 2009; Hoffmann, 2013). The population declines and isolation experienced by many native breeds following the expansion of modern agriculture (Taberlet et al., 2008, 2011) typically occur because they are less productive – in terms of, e.g., milk, wool, or meat production – than the commercial breeds. The native breeds may be adapted to a more stringent environment and climate, such as that of high mountains and northern coastal areas that are relatively marginal for agriculture. Hence, their commercial disadvantage might protect important genetic variants that promote survival in harsher climates (e.g., Hoffmann and Parsons, 1997; Joost et al., 2007). Preservation of genetic diversity in native breeds may have key evolutionary applications (Kantanen et al., 2000; Taberlet et al., 2008), including adaptation to climate change and in promoting sustainable agriculture (Hoffmann, 2013).

Environmental conditions for domestic animals are typically benign, increasing the probability of survival for crosses that might not otherwise survive in the wild. However, where livestock are maintained for free-roaming grazing and landscape management, their survival under difficult environmental conditions may be paramount. For a thorough examination of inbreeding and outbreeding it is necessary to study the entire life cycle and not focus on any single component of fitness (Edmands, 2007), and also to consider these effects on organisms in their natural environment (Kristensen et al., 2008; Enders and Nunney, 2012). Furthermore, Edmands (2007) highlights the critical importance of understanding how hybridization affects the generations beyond F2 and the initial back-crosses, although any such effects will be ameliorated to some extent by ongoing selection against poorly adapted F2 genotypes (Weeks et al., 2011). Genetic rescue may be possible when the only available donors are from other inbred populations, and reciprocal translocations or gene flow between such populations may provide an important short-term measure for conservation (Heber et al., 2013). However, inbreeding and outbreeding depression may occur at the same time and their effects can be difficult to distinguish, especially in managed populations (Edmands, 2007 and references therein). These findings merit additional attention for endangered species of wild and domestic species, and the latter may provide important opportunities for experimental and controlled study with benefits for both wildlife and livestock at risk.

## HOW CAN GENOMICS BENEFIT CONSERVATION OF LIVESTOCK BREEDS AND WILD POPULATIONS?

Current management guidelines for populations at risk frequently emphasize genetic uniqueness over genetic diversity (Funk et al., 2012). Such practices may need review, as trade-offs between genetic diversity and genetic uniqueness has been observed (Coleman et al., 2013). For example, the endangered dwarf galaxias *Galaxiella pusilla* populations reported to be genetically most unique also have the least amount of genetic variation when assessed with microsatellite genetic markers (Coleman et al., 2013). This relationship between uniqueness and diversity may be a general observation in small and threatened populations where genetic structure is strongly affected by genetic drift. Prioritizing populations that are genetically unique may therefore, at times, decrease overall genetic diversity and, accordingly, reduce evolutionary potential. These issues have been recognized more widely in genetic management of domestic species (Barker, 2001; Caballero and Toro, 2002).

Genomic tools can contribute to genetic resource management through accurate estimation of genetic uniqueness and control of inbreeding (Li et al., 2011; Amador et al., 2014; Pertoldi et al., 2014). Breeding schemes based on information from commercial single nucleotide polymorphism (SNP) chips for example can be used to select efficiently against deleterious alleles in a population and/or select for increased heterozygosity in genes of adaptive significance provided these can be identified (for an example see, Marcos-Carcavilla et al., 2010). Thus revision of breeding plans based on genome-wide study of variation within populations is now a practical option. For example Pertoldi et al. (2014) showed how genome-wide SNP data can be used to design breeding programs aiming at reducing the loss of genetic variability within a small population of Danish cattle by prioritizing matings between individuals with relatively low pairwise identity-by-state.

Another element of genetic rescue could involve adaptive management toward climate change (Aitken and Whitlock, 2013), where native breeds well-adapted to local conditions such as high precipitation (Joost et al., 2007) might provide genetic material to commercial breeds and isolated populations of the same or similar breeds. This may be necessary in view of future climate change, where adaptations to factors such as hot, arid, and saline conditions (Hoffmann, 2010; Hoffmann et al., 2013) may be increasingly essential for survival. Caballero and Toro (2002) developed an approach that balances genetic diversity against uniqueness. However, this approach does not account for the fact that some unique populations may contain private alleles of adaptive value, which can arise as a consequence of either random genetic drift or strong selection. Genomic tools that permit identification of such genetic variants will therefore be highly valuable for identifying populations, and individuals within populations, of special importance for long-term conservation. Using genomic selection, these genes can be introduced very rapidly into populations.

Incorporating more individuals in breeding management will augment Ne, and candidate profiles should be evaluated with consideration to long-term evolutionary potential. This could involve weighing genetic diversity against uniqueness (Coleman et al., 2013); in some cases donor individuals may have overall low genetic diversity, but carry unique genetic variants that can benefit the more genetically diverse recipient population for specific traits. This is relevant if genetic variability in a commercial breed is considered for augmentation from a small local breed. In the short term, donor individuals may not have the highest breeding values for production traits, but in a broader perspective they could contribute valuable material with respect to increased robustness when exposed to diseases and environmental variability associated with climate change. Here, genome-wide profiles can assist in selecting individuals with the desired features of the local breed such as alleles associated with parasite resistance (Coltman et al., 2001), while matching the purpose of the commercial breed as closely as possible (production of, e.g., milk, meat, or wool).

An important difference between wild and domestic species is that not only fitness – survival, growth, fecundity – but also production and output in economic terms will be important in domestic species. That is, natural selection for independent survival in the local environment is traded for traits such as high yield of milk and meat that may be unsustainable under natural conditions [an extreme example is selection for cattle muscle mass necessitating high frequencies of Cesarean sections, e.g., Pirottin et al. (2005)]. Native and relatively naturally selected breeds may contribute genetic material to commercial breeds, and these could in turn contribute genetic variation to native breeds. The direction(s) and scale of gene flow will depend on the specific conservation breeding objectives, such as avoiding extinction of a small native breed, or increasing the frequency of disease resistance-associated alleles in a large commercial herd.

Genome-wide profiles in the form of SNP markers permit investigation of neutral and functional genes, providing insight into a broader range of evolutionary processes (e.g., Rice et al., 2011). This can help optimize breeding management in the form of detailed information on variation present within a herd, across a given breed, or within a species. A small native breed may have few or no unrelated individuals and may be considered for limited cross-breeding with a more variable breed to improve the probability of persistence. Genomics offers a tool to help screen and prioritize contributions, where traditional use of phenotypic information can be combined with accurate data on individual relatedness and genetic diversity.

The highly managed and small Ne of many domestic breeds provide valuable learning opportunities to help bridge the gap between laboratory model organisms and wild species in understanding the strength of selection in small populations. One example is work to identify the chondrodystrophy (dwarfism) locus in California condor which is being aided by comparisons with the DNA sequence of the domestic chicken (Romanov et al., 2009). Such examples can benefit conservation of endangered wildlife and domestic species, and inform future work on model organisms.

Another example is the work currently underway to cross the dog breeds Lundehund and Buhund to augment the genetic diversity and conservation (including health status) of the Lundehund. Hybrid pups were born in 2014^2^, and will be evaluated with respect to factors such as morphology and behavior. Careful selection of future animals used for breeding should be possible through genomic analyses of hybrid profiles, by following backcrosses between F1-hybrids and Lundehund. An important objective will be to retain physical characteristics of the Lundehund breed whereas there is urgent need to reduce the prevalence and severity of the breed’s gastrointestinal problems. The methods proposed by Amador et al. (2014) on the use of genomic selection to recover the original genetic background from hybrids may be highly applicable in this respect. Identification of genes involved in similar gastrointestinal disorders is advancing for humans (see, e.g., McGovern et al., 2010 on ulcerative colitis and Crohn’s disease). Even though other genes may be implicated in gastrointestinal diseases of dogs, breeding individuals may be selected that contribute new genetic variation to the Lundehund for the genome regions known to be affected in humans.

With domestic species it is relatively easy to perform controlled experimental breeding and evaluate offspring characteristics, helping to assess the consequences of mixing two breeds, and optimizing strategies for cross-breeding. Thus genomic data can help expand conservation management of farm animals by permitting a careful, adaptive process with a long-term perspective whereby breeds are explicitly considered as an evolutionary work-in-progress (**Box 2**).

**BOX 2. Insights/tools for genetic management of small populations.**

• Many hereditary diseases are caused by recessive deleterious alleles. Genomic tools are useful for identifying the causal mutation and for detection of carriers (heterozygotes). *Example:* Identification and efficient selection against the mutation causing complex vertebral malformation in Holstein dairy cattle ( Thomsen et al., 2006; Whitlock et al., 2008).
• Cryopreservation of biological material including semen, oocytes, and embryos is possible for most livestock species (Mara et al., 2013) but not developed for most species of conservation concern. *Perspectives:* The use of frozen semen or embryos in breeding plans for small populations can decrease inbreeding and increase genetic variation (Sonesson et al., 2002; Woelders et al., 2012).
• Genomes of most species can now be sequenced for relatively reasonable costs (Hayden, 2014). *Perspectives:* Novel applications of genomic data in domestic and non-domestic populations include: (1) genome-wide profiles comprising neutral and adaptive genetic variation enabling selection for specific adaptive alleles and genetic variation, (2) estimates of Ne that are accurate and comparable, and (3) description of the demographic history of populations.
• Inbreeding depression is a trait, population and environment-specific phenomena. *Perspectives:* Generalizations are problematic but simple guidelines suggest that Ne >50 is sufficient to avoid significant inbreeding depression in livestock (Meuwissen and Woolliams, 1994) whereas this number likely needs to be doubled in wild populations (Frankham et al., 2014).
• The effective population size, Ne, is below 500 in the vast majority of livestock breeds and threatened species in nature. *Perspectives:* Long-term evolutionary change is likely constrained in these populations. Genetic rescue through hybridization is a tool to increase Ne and genetic variance and reduce inbreeding in small natural populations (Hogg et al., 2006) and domestic breeds (Schaeffer et al., 2011).

## FUTURE OUTLOOK

The above considerations indicate that genomic data provide highly useful information about the applicability and likely success of strategies aimed at increasing and maintaining the adaptability of threatened breeds and natural populations. By providing information on the genetic architecture of complex traits, these approaches provide tools that are changing the way we approach conservation of genetic variation in livestock and natural populations. Whereas genomics is not a magic bullet that will instantly permit full overview of the genome and the interactions among its parts, the use of an increased number of genetic markers through next generation sequencing approaches will augment the accuracy of estimating diversity and population demographic parameters of conservation relevance (Allendorf et al., 2010; Shafer et al., in press). Furthermore it opens up the possibility to screen individuals and populations for adaptive loci and use genomic information to guide breeding decisions including which populations and individuals to use in genetic rescue programs (Amador et al., 2014; Toro et al., 2014). Genome-scale data will also be increasingly used to document the demographic impact resulting from genetic rescue programs. In one example Miller et al. (2012) showed that migrant alleles (from translocated individuals) increased over time in an insular population of bighorn sheep (*Ovis canadensis*). More generally, Amador et al. (2014) provide a framework on how to use genomic selection for recovery of original genetic background from hybrids. At this early stage methods still need to mature, pipelines for dealing with assembly and annotation in non-model organisms need further development, and clear examples of practical applications need to be disseminated to practitioners (Shafer et al., in press).

For conservation of livestock breeds and small populations in nature, we advocate that long-term genetic and phenotypic monitoring is needed, and based on such data management decisions are taken (**Box 1**). Current and past performance of a population may represent poor predictors of future performance due to factors such as genotype by environment interactions, inbreeding, genetic drift, changed breeding objectives, and environmental changes. In the long run genetic variation will be depleted in populations with small Ne. These populations will become constrained in their evolutionary responses and are likely to go extinct in environments that change rapidly and may become stressful, although the experience with livestock suggests that as long as environments are relatively constant phenotypic changes remain possible under strong selection. Nevertheless, threatened populations should not be regarded as museum specimens, and an increased focus on Ne and genetic variation in populations of conservation concern should help promote the potential for adaptive evolution. For small and threatened breeds one way to achieve this is through limited cross-breeding and active use of genomic data to guide breeding decisions.

## Conflict of Interest Statement

The authors declare that the research was conducted in the absence of any commercial or financial relationships that could be construed as a potential conflict of interest.

## Footnotes

1 http://lundehund.no/

2 http://lundehund.no/om-norsk-lundehund-klubb/ras
